# Case report: Novel insights into hemorrhagic destruction of the brain, subependymal calcification, and cataracts disease

**DOI:** 10.3389/fped.2023.1178280

**Published:** 2023-09-13

**Authors:** Tameemi Abdallah Moady, Marwan Odeh, Ayalla Fedida, Zvi Segal, Maayan Gruber, Moshe Goldfeld, Limor Kalfon, Tzipora C. Falik-Zaccai

**Affiliations:** ^1^ Institute of Human Genetics, Galilee Medical Center, Nahariya, Israel; ^2^ Azrieli Faculty of Medicine, Bar Ilan University, Safed, Israel; ^3^ Ob/Gyn Ultrasound Unit, Galilee Medical Center, Nahariya, Israel; ^4^ Department of Ophthalmology, Galilee Medical Center, Nahariya, Israel; ^5^ Department of ENT, Galilee Medical Center, Nahariya, Israel; ^6^ Department of Radiology, Galilee Medical Center, Nahariya, Israel

**Keywords:** *JAM3/JAM-C*, HDBSCC, congenital cataracts, auditory neuropathy, brain hemorrhages, brain calcification

## Abstract

**Introduction:**

Pathogenic variants of the junctional adhesion molecule 3 (*JAM3/JAM-C*; OMIM#606871) is the cause of the rare recessive disorder called hemorrhagic destruction of the brain, subependymal calcification, and cataracts (HDBSCC, OMIM#613730) disease. A similar phenotype is universal, including congenital cataracts and brain hemorrhages with high mortality rate in the first few weeks of life and with a poor neurologic outcome in survivors. We aim to describe and enlighten novel phenotype and genotype of a new patient and review the literature regarding all reported patients worldwide.

**Case report:**

We report the case of a prenatal and postnatal phenotype of a new patient with a novel pathogenic loss-of-function variant in *JAM3*, who presented prenatally with cataracts and brain anomalies and postnatally with brain hemorrhages, failure to thrive (FTT), progressive microcephaly, recurrent posterior capsule opacities, and auditory neuropathy.

**Discussion:**

This study enlightens novel possible functions of *JAM3* in the normal development of the brain, the ocular lenses, the auditory system, and possibly the gastrointestinal tract. This study is the first to report of cataracts evident in as early as 23 weeks of gestation and a rare phenomenon of recurrent posterior capsule opacities despite performing recurrent posterior capsulectomy and anterior vitrectomy. We suggest that auditory neuropathy, which is reported here for the first time, is part of the phenotype of HDBSCC, probably due to an endothelial microvasculature disruption of the peripheral eighth nerve or possibly due to impaired nerve conduction from the synapse to the brainstem.

**Conclusions:**

Prenatal cataracts, brain anomalies, FTT, and auditory neuropathy are part of the phenotype of the HDBSCC disease. We suggest including *JAM3* in the gene list known to cause congenital cataracts, brain hemorrhages, and hearing loss. Further studies should address the auditory neuropathy and FTT phenomena in knockout mice models. We further suggest performing comprehensive ophthalmic, audiologic, and gastroenterologic evaluations for living patients worldwide to further confirm these novel phenomena in this rare entity.

## Introduction

Pathogenic variants of the *JAM3/JAM-C* gene were first reported in 2010 (1) and 2013 (2) to cause the rare recessive disorder called hemorrhagic destruction of the brain, subependymal calcification, and cataracts (HDBSCC, OMIM#613730) by mimicking an intrauterine toxoplasma, rubella, cytomegalovirus, Herpes simplex, and HIV (TORCH) infection. All the reported patients of this disease presented with congenital cataracts and brain hemorrhages with high mortality rate in the first few weeks of life. A poor neurodevelopmental outcome was noted in survivors, with progressive microcephaly, generalized spasticity, hyperreflexia, and seizures. A total of 13 patients have been reported worldwide (1–4); demographic details, phenotype, and genotype of patients reported in the literature are summarized in Table 1.

**Table 1:** Clinical and molecular summary of all reported patients to date.

Reference	Descent	Patient number	Gender	Age at the time of published paper	Other features	Genotype
(1)	UAE^a^	1	M	Died at 9 days	Hepatomegaly, cardiac anomaly	c.747 + 1G > T
	UAE	2	F	Died after birth	Short rib, polydactyly type III, RA	c.747 + 1G > T
	UAE	3	M	3 years 4 months	Seizures, small phallus, undescended testis	c.747 + 1G > T
	UAE	4	F	1 year 2 months	Seizures, thrombocytopenia while bacterial meningitis	c.747 + 1G > T
	UAE	5	M	Died at 2 months	RA	c.747 + 1G > T
	UAE	6	F	6 years 1 month	Seizures, RA	c.747 + 1G > T
(2)	Turkey	7	F	Died at 2 months	Seizures	c.656G > A
	Afghani	8	M	Died at 2 weeks	RA, white matter abnormalities	c.346G > A
	Afghani	9	M	Died at 5 days	Abnormal EEG^b^, RA	c.346G > A
	Morocco	10	M	Died at 10 days		c.2T > G
	Morocco	11	F	Died at 39 days	Abnormal EEG, thin CC	c.2T > G
(3)^c^	Spain	12	F	Died at 43 days	Seizures	c.2T > G
(4)	Italy	13	M	24 months	Myocardial hypertrophy, FTT, hypoplasia of cerebellar vermis	c.690T > G
Current patient	Israel (Bedouin)	14	M	30 months	Abnormal EEG and seizures, auditory neuropathy	c.745dup

RA, renal anomaly.

^a^ United Arab Emirates.

^b^ EEG, electroencephalogram study.

^c^ Poster abstract at *J Matern Neonatal Med*. (2016).

*JAM3/JAM-C* is a transmembrane 43 kDa glycoprotein and a member of the immunoglobulin (Ig) superfamily. The *JAM* family (*JAM1/JAM-A*, *JAM2/JAM-B*, *JAM3/JAM-C*, *JAM4*) and related proteins (such as occludin) participate in the assembly and maintenance of tight junctions, angiogenesis, vascular permeability, polarity, and leukocyte trans-endothelial cell migration and are involved in multiple inflammatory processes such as pulmonary inflammation. Dynamic *JAM3* trafficking and degradation are necessary for junctional remodeling during cell migration and angiogenesis. In addition, *JAM3* seems to play a role in tumor genesis and is therefore a biological biomarker in different cancers (5–9).

Knockout *JAM3* mice models reveal that *JAM3* is critical for the differentiation of spermatids (10), integrity of the myelin sheath, nerve conduction and motor function (11), lens development (12, 13), and esophagus innervation (14), but *JAM3* probably has no role in heart morphology and function (15). *JAM3*-deficient mice show megaesophagus, failure to thrive (FTT), jitteriness, multilobar pneumonia, brain hemorrhages, and communicating hydrocephalus (16).

## Case presentation

**Figure 1:**
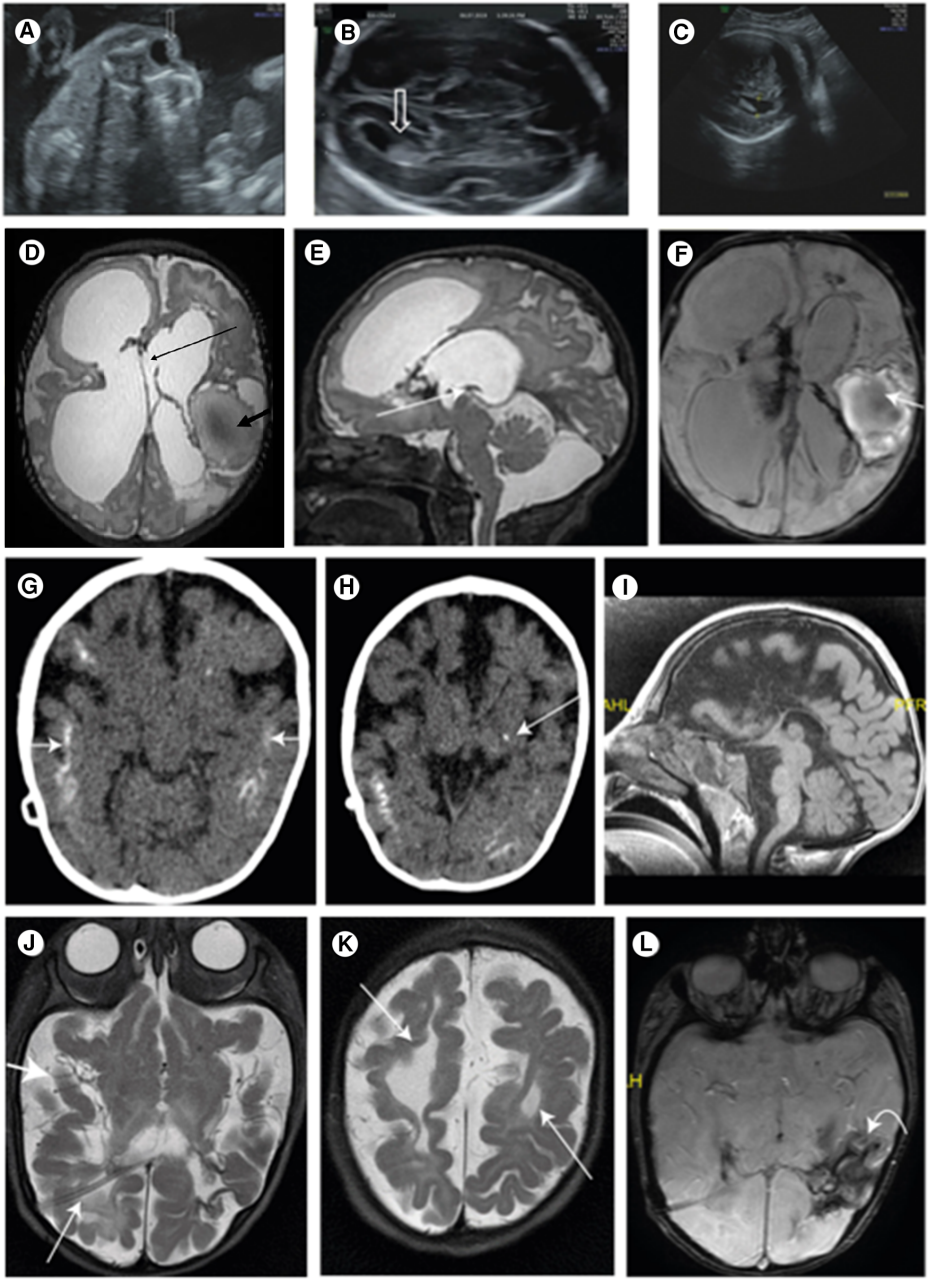
(**A**) Central echogenicity of the lens (arrow) indicating congenital cataract at 23 WG; (**B**) normal lateral ventricle (arrow) at 23 WG; (**C**) ventriculomegaly of 13.4 mm at 34 WG indicating the progressive nature of the disease; (**D**) T2 FSE axial brain. Large ventricles: Right frontal porencephalic cysts bold arrows: hemorrhagic cystic malacia. Interhemispheric cyst connecting the lateral ventricles thin arrow; (**E**) sagittal brain T2 fast spin echo (mri sequence name) (FSE) thin arrow: closed aqueduct; (**F**) swan axial brain thin arrow: hemorrhagic cavity; blood product in the lateral ventricles; (**G**) brain CT scan without contrast media: after VP shunt. Bilateral, extensive, cortical calcifications (white arrows); (**H**) a spot of basal nuclei calcification on the left (white arrow); (**I**) MRI after VP shunt. Mid sagittal. The corpus callosum is not seen; (**J**) MRI after VP shunt. Shunt in the right side (RT) lateral ventricle (thin arrow). The third and lateral ventricles are very small, and the subarachnoid spaces are enlarged (thick arrow), due to decrease of the pressure; (**K**) the cystic malacia changes decreased in size (thin arrow); (**L**) intracranial blood products are more clumped (curved arrow).

The patient is the first child of a consanguineous couple. The prenatal follow-up included normal nuchal translucency and first-trimester biochemical screening tests. The fetal sonography at 14 weeks of gestation (WG) was normal except for a single umbilical artery. The sonography at 23 WG demonstrated bilateral centric cataracts and echogenicity in the lateral brain ventricles (Figures 1A,B). The brain fetal sonography at 30 WG showed dilated lateral ventricles up to 13 mm, irregularity of ventricle walls, and suspected underdeveloped sulci (Figure 1C).

The serologic tests for TORCH and parvovirus were negative. The family refused to undergo prenatal molecular diagnosis.

The infant was spontaneously delivered at 37 WG with Apgar scores of 9/10, birth weight of 2.4 kg (10th percentile), and head circumference of 32 cm (6th percentile) at birth. The physical examination at birth was normal except for bilateral central dense nuclear cataracts and retrognathia.

The brain sonography at Day 4 of life revealed thin corpus callosum (CC), dilated lateral ventricles, hemorrhage in the left thalamus, and the presence of mega cisterna magna. Visceral and cardiac sonographies were normal.

Other blood tests such as biochemistry, hematology, and metabolic workup were also normal.

At age 18 days, the patient’s brain sonography showed new foci of bleeding in the brain parenchyma and ventricles with periventricular leukomalacia (PVL). The lumbar puncture showed a yellowish cerebrospinal fluid (CSF): presence of hypoglycorrhachia (21.7 mg/dl) and low lactate level (1.82 mg/dl), high total protein level (236 mg/dl), white blood cell (WBC) count of 10, low red blood cell (RBC) count, and normal amino acid profile.

The brain MRI at age 24 days showed an extensive brain parenchymal damage and cystic malacia changes, some communicating with the ventricular system and large hemorrhagic cavities. The lateral and third ventricles were enlarged. The CC was not depicted. The aquaeductus was stenotic, and no flow voids were visible. A large infra-tentorial fluid collection was depicted (Figures 1D–F). A ventricular peritoneal (VP) shunt was inserted urgently on Day 25.

The CT scan at age 12 months showed very small third and lateral ventricles and an enlargement of the subarachnoid spaces consistent with brain atrophy. Extensive brain calcifications were observed, mainly cortical and subcortical and less calcification in the basal ganglia (Figures 1G,H).

The brain MRI at age 5 months showed the absence of the CC (Figure 1I) and the Probst bundles, suggesting that the CC was destroyed. Smooth gyri in the right frontal hemisphere and white matter hypoplasia were observed. Blood products in lateral ventricles and in the third ventricles and parenchymatic hemorrhage in the left parietal and temporal hemispheres were more clustered (Figures 1J–L).

A recent brain MRI at age 30 months was extremely abnormal but stable.

At 30 months, a severe growth delay was noted: weight and height were 2.7 standard deviation (SD) below normal for age and gender, and a severe progressive microcephaly was observed (−4.1 SD). Jitteriness, episodes of severe restlessness, and intractable seizures were documented by an abnormal EEG result. The patient’s development was severely delayed, and no social interactions were seen, with severe generalized hypertonia and opisthotonos, hyperreflexia, and a positive Babinski sign.

**Figure 2:**
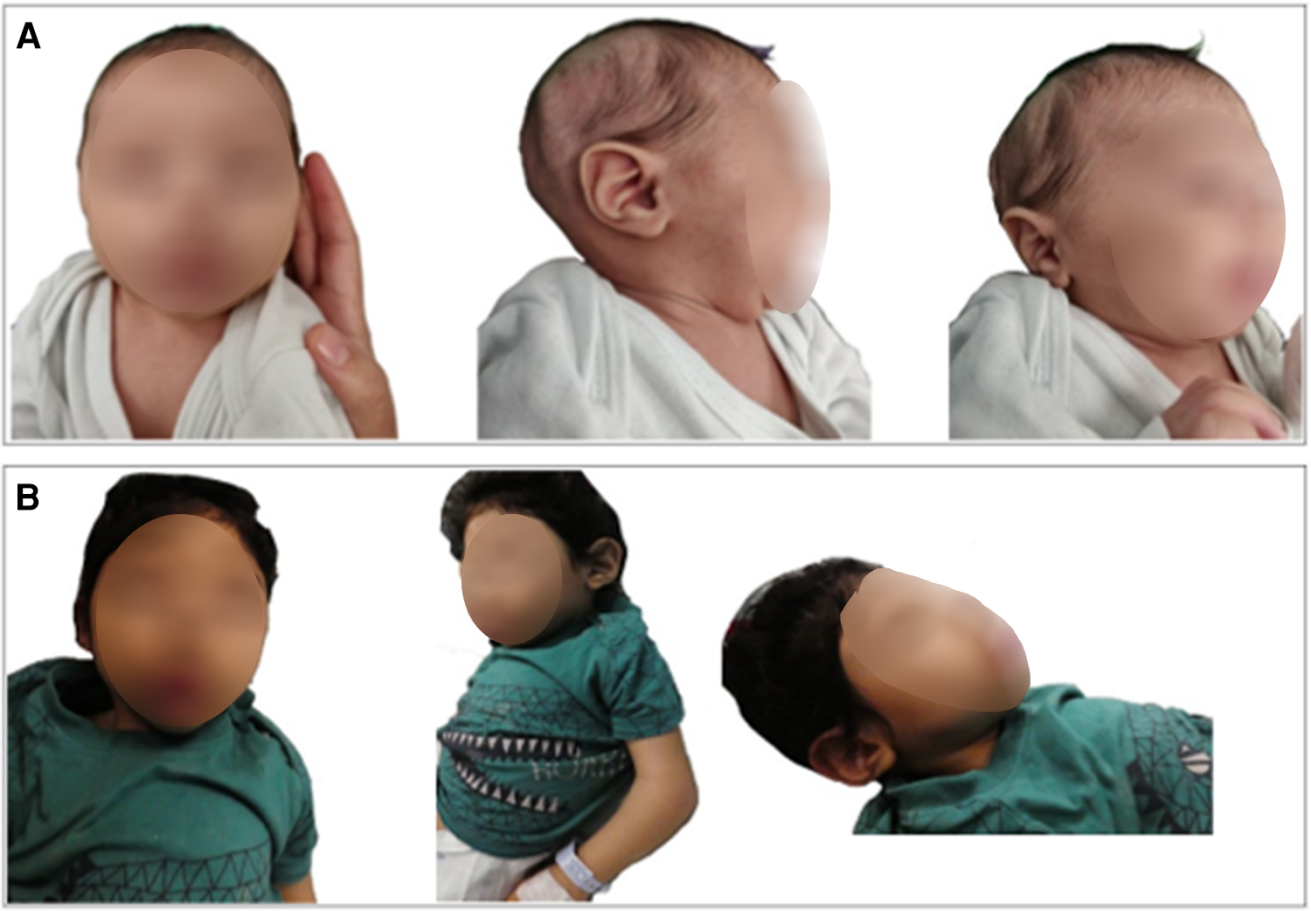
Our patient at 7 months of age (**A**) and 30 months (**B**) with evolving dysmorphic features which are now more prominent. Photophobia, high forehead with low anterior hair line, thin nasal bridge, synophrys with high arched eyebrows, epicanthus with down slanting eyes, micro retrognathia, posteriorly rotated ears, puffed cheeks, and short neck with abundant skin folds.

The unique dysmorphic features that became more prominent with time included high forehead with low anterior hair line, thin nasal bridge, synophrys with high arched eyebrows, epicanthus with upper slanting eyes, posteriorly rotated low-set ears, puffed cheeks with tented mouth, short neck with abundant skin folds, three hair whorls, tapering of fingers, and abnormal fat distribution (Figures 2A,B).

The newborn hearing screening conducted at age 2 days by otoacoustic emission (OAE) was normal bilaterally. The comprehensive diagnostic auditory evaluation was completed at age 3 months using transient evoked OAE (TE-OAE) and auditory brain stem response (ABR). TE-OAE results were within normal range while ABR did not record any conclusive electrical wave compositions. The hearing thresholds were suggestive of profound hearing loss, and the clinical combination of normal hair-cell function without normal brainstem wave composition was consistent with auditory neuropathy spectrum disorder (ANSD).

The ophthalmic examination was normal except for the cataracts. At 6 weeks, the neonate underwent lens aspiration with posterior capsulotomy and anterior vitrectomy in his left eye, and a month later in the right eye. After 1 month, he was re-operated since severe central posterior opacity like dense Elschnig pearls developed in his both eyes. Two months later, re-opacification has occurred; the thick and stiff tissue could not be removed by vitrectomy, and the membrane was excised. The visual axis remained clear.

### Molecular genetic workup

The chromosomal microarray analysis (CMA) was normal.

Considering the unique features of cataracts and brain hemorrhages and consanguinity, Sanger sequencing of *JAM3(NM_032801)* was performed according to established protocols. Sequence data (coding region +30 flanking bps) were analyzed using NCBI Nucleotide Blast (https://blast.ncbi.nlm.nih.gov/Blast.cgi) by alignment to GRCh38 assembly, revealing a novel pathogenic frameshift homozygous variant: c.745dup, p. (Val249Glyfs*28), according to the American College of Medical Genetics and Genomics (ACMG) guidelines. Both parents were heterozygous (Supplementary Figure S1).

A panel of 300 genes related to hearing loss revealed no pathogenic/likely pathogenic genetic variants that could possibly explain auditory neuropathy.

## Discussion

*JAM3* biallelic loss-of-function variants cause the rare HDBSCC disease in humans (OMIM#613730). We report the prenatal and postnatal findings of a new patient with HDBSCC alive at 30 months. We expand the phenotype to include prenatal cataracts with recurrent posterior capsule opacities, growth failure, progressive microcephaly, brain anomalies, dysmorphic features, jitteriness and restlessness, and auditory neuropathy, which is reported here for the first time. We did not document thrombocytopenia, hepatomegaly, and renal or cardiac anomalies in our patient, or other visceral organ involvement previously reported in other patients. The unique combination of cataracts and brain hemorrhages are almost pathognomonic for HDBSCC after excluding TORCH infection. Sanger sequencing of *JAM3* revealed a pathogenic novel loss-of-function variant in our patient. Some *JAM*-related family genes cause an overlapping partial phenotype in addition to other genes such as heterozygous defects in *COL4A* (collagen type IV, alpha1) gene (MIM-120130), resulting in microangiopathy, brain calcifications, and porencephaly (OMIM#175780), with some cases reporting cataracts (17).

*JAM3* is highly expressed in platelets (18); nevertheless, the brain multifocal hemorrhages are probably not due to platelet dysfunction but rather to the *JAM3* role in cerebrovascular endothelium. No evidence of systemic bleeding diathesis is noted in any of the reported patients. Thrombocytopenia is documented in one patient with bacterial meningitis (1). Brain calcification is demonstrated in all reported patients similar with the current patient. Idiopathic basal ganglia brain calcifications 8 (OMIM# 618824) are reported in *JAM2* gene defects (MIM#606870), which is part of the *JAM* family but with no evidence of cataracts or brain hemorrhages in these patients (19, 20).

Pseudo-TORCH syndrome 1 (OMIM#251290) is an autosomal recessive disorder due to the defective *OCLN* gene (MIM#602876) encoding occludin, which is another *JAM* family-related protein. This gene defect possibly causes brain calcifications, cortical dysgenesis, severe developmental delay often with hepatic and renal dysfunction, and thrombocytopenia, but with no evidence of brain hemorrhages or cataracts (21, 22).

In a report by Mochida et al. (1), brain anomalies such as cortical dysgenesis were not detected in the patients. In another report by Akawi et al. (2), one patient had thin CC, and another patient had white matter abnormalities, both of which were demonstrated in our patient. Furthermore, hypoplasia of cerebellar vermis was documented in one patient, in addition to PVL at age 1 day (4). We would like to emphasize that the brain morphology was abnormal in our patient's prenatal sonography at 30 WG, suggesting the role of *JAM3* in normal brain development. In our patient, the atrophy and wide sulci demonstrated at 5 months of age are probably secondary to tissue damage, cystic degeneration, and liquefaction after hemorrhagic destruction. The tissue destruction and atrophy are followed by progressive microcephaly. Thin CC, cortical dysgenesis, and auditory neuropathy could be explained by the important role of *JAM3* in Schwann cells, myelin sheath, and neural migration (11). The central nervous system (CNS) phenotype in *JAM3* defects is not solely due to destructive hemorrhages but also related to several key functions of *JAM3/JAM-C*, which participates in the assembly and maintenance of tight junctions in the CNS. Furthermore, a communicating hydrocephalus has occurred in mice knockout models for *JAM3*, suggesting that *JAM3* has an additional function in permeability and CSF homeostasis; thus, the CNS phenotype is variable due to several interactions and functions of *JAM3*.

This is the first report of auditory neuropathy in an HDBSCC patient. ANSD is a subtype of sensorineural hearing loss, which is characterized by a disruption of the synchronization of transmitting excitation along the auditory pathway (desynchrony). It is reported to be the cause of hearing loss in about 10% of children with severe hearing loss (23). Its diagnosis is often delayed since newborns with this condition typically pass the OAE hearing screening that specifically examines the outer hair cells of the inner ear (24), which are preserved in ANSD. Therefore, the diagnosis is confirmed later by the combined results of OAE and ABR. Pathophysiology is based on the loss or dysfunction of the inner hair cells and/or more internal structures. An underlying genetic cause may be determined in about 40% of children with ANSD (25). In our patient, the next generation sequencing (NGS)-based hearing loss gene panel did not detect any variants to explain the hearing loss. We suggest that auditory neuropathy is part of the phenotype of HDBSCC, probably due to endothelial microvasculature disruption of the peripheral 8th nerve or possibly due to impaired nerve conduction from the synapse to the brainstem. More studies are warranted to investigate this phenomenon in knockout mice models, and we recommend a comprehensive audiologic evaluation for alive patients worldwide to further confirm this novel phenomenon as part of this syndrome.

In our patient, cataracts were evident as early as 23 WG. In an Italian patient (4), cataracts were also suspected prenatally, suggesting the role of JAM3 in early genesis of the lenses. Re-opacification of the lens capsule is very rare (26), especially if the posterior capsule or anterior hyaloid does not exist. Here, we report an extremely rare situation of two re-opacification occurrences post-posterior capsulotomy with anterior vitrectomy in congenital cataracts. Opacification of the posterior surface of the intraocular lenses may be another mechanism of re-opacification in the absence of posterior capsule or anterior hyaloid, however in our patient, the eyes were aphakic. Uveitis or inflammatory reaction may induce re-opacification, but no such inflammation was observed in serial follow-up. A new study regarding lens development in knockdown mice showed abnormal lens morphology and defective degradation of nuclei and organelles in lens fiber cells. Cell death and abnormal lens development was accompanied by the activation of unfolded protein response (UPR) (13).

In a recent report of an Italian patient suspected to have prenatal cataracts in addition to severe growth delay, microcephaly, seizures, and cardiac hypertrophy (4), the patient had feeding difficulties necessitating gastrostome insertion. Our patient was restless and presented FTT despite normal caloric intake. Interestingly, knockdown mice show FTT and megaesophagus. This might emphasize the roles of *JAM3* in permeability and innervation since it is also present in vascular smooth muscle cells and not only in brain endothelial cells. Further studies of *JAM3* functions in the gastrointestinal tract are warranted.

Our report is limited to one patient diagnosed with an ultra-rare disease. It is challenging to confirm the relationship between the novel phenotypes (auditory neuropathy, FTT, and early onset cataracts) described here to the HDBSCC syndrome and the *JAM3* genetic variant. Furthermore, patients who are the born of a consanguineous marriage may have more than one disease; therefore, we cannot rule out the existence of several distinct diseases in this patient such as a distinct hearing defect. However, considering normal NGS hearing loss panel, this possibility is less reasonable. Further studies are warranted in mice and living patients to confirm these phenomena.

To date, we have followed up our patient for 3 years. During this time, the parents decided to undergo prenatal diagnosis in their succeeding two pregnancies, and two healthy babies were born. The molecular diagnosis enabled carrier screening and identification of couples at risk among the extended family (Chamulah).

## Conclusions

This report enlightens the novel possible functions of *JAM3* in the normal development of the brain, ocular lenses, and the auditory system function, and possibly gastrointestinal tract. We recommend further studies addressing the gastrointestinal and ocular systems as well as auditory function in mice models and an audiologic assessment of living patients to further confirm these novel phenotypes in HDBSCC. We suggest including *JAM3* in the gene list known to cause prenatal/congenital cataracts and hearing loss. Molecular workup might confirm this devastating diagnosis earlier and facilitate better decision-making and accurate genetic counseling for families.

## Data Availability

The original contributions presented in the study are included in the article/supplementary material, further inquiries can be directed to the corresponding author/s.
