# Supplementary material for: Case report: Novel insights into hemorrhagic destruction of the brain, subependymal calcification, and cataracts disease
Source: Front Pediatr. 2023 Sep 13;11:1178280. doi: 10.3389/fped.2023.1178280 (PMC10534027; doi:10.3389/fped.2023.1178280)

SUPPLEMENTARY MATERIAL

Case Report: Novel insights into Hemorrhagic destruction of the brain, sub ependymal calcification and cataracts (HDBSCC) disease

Tameemi Abdallah Moady MD, Odeh Marwan MD, Ayalla Fedida PhD, Zvi Segal MD, Maayan Gruber MD, Moshe Goldfield MD, Limor Kalfon PhD, Tzipora C Falik-Zaccai MD

Corresponding author:
Professor Tzipora C Falik-Zaccai, MD
Tel: 972-50-7887941
Fax: 972-4-9107553
Email: falikmd.genetics@gmail.com

Supplementary Figure 1: Sanger sequencing illustrating the variant c.745dup p.(Val249Glyfs*28) in JAM3. Electropherograms demonstrating homozygous reference allele (Healthy control, upper panel) heterozygous allele (Parent, Middle panel) and homozygous alternate allele (Proband)

Healthy control

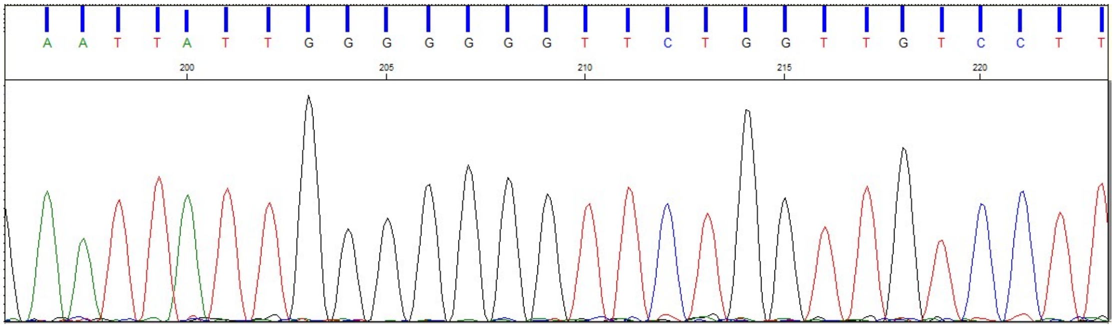

Parent (carrier)

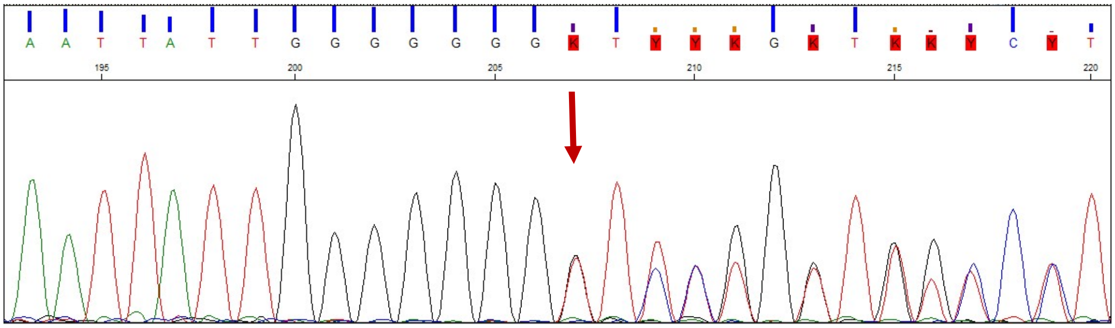

Proband

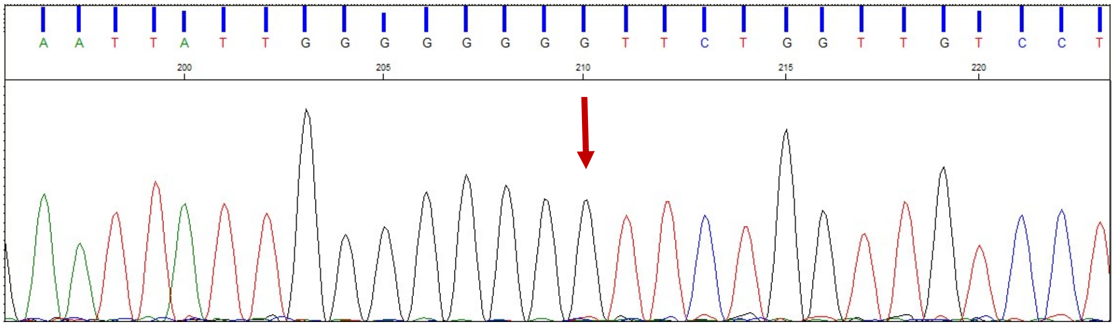

Supplement: Supplementary file 1 [file Image1.pdf]
